# Impact of ‘street’ benzodiazepines on drug‐related deaths in England, Wales and Northern Ireland

**DOI:** 10.1111/dar.13979

**Published:** 2024-11-28

**Authors:** Kirsten L. Rock, Anca Frinculescu, Trevor Shine, Nicola J. Kalk, Caroline S. Copeland

**Affiliations:** ^1^ Institute of Pharmaceutical Science, King's College London London UK; ^2^ TICTAC Communications Limited London UK; ^3^ Department of Analytical, Environmental and Forensic Sciences King's College London London UK; ^4^ South London and Maudsley NHS Foundation Trust London UK; ^5^ Addictions Department Institute of Psychology, Psychiatry and Neuroscience, King's College London London UK; ^6^ National Programme on Substance Abuse Deaths London UK

**Keywords:** benzodiazepines, diazepam, forensic toxicology, novel benzodiazepines, street benzodiazepines

## Abstract

**Introduction:**

‘Street’ benzodiazepines (BZD) are structurally and pharmacologically related to BZDs licensed for human use. In this study we investigated how street BZDs contribute to overall BZD use and death prevalences in England, Wales and Northern Ireland.

**Methods:**

Data were analysed from deaths reported to the National Programme on Substance Use Mortality with post‐mortem BZD detections (1999–2021), BZDs seized from music festivals (2017–2021) and drug samples with BZD detections submitted to Welsh Emerging Drugs and Identification of Novel Substances (WEDINOS) (2017–2021).

**Results:**

About 14,837 deaths with BZD detections were identified, with polydrug use evident in 99.3% of cases (*n* = 14,733/14,837). Deaths following BZD use increased by over 200% from 2010 (*n* = 556) to 2020 (*n* = 1245). Most BZD detections were of those available via NHS prescription (96.2%), although in most cases (61.9%) the BZD—majority diazepam (77.3% of detections)—had been illicitly sourced. While street BZD deaths represented only 8.5% of overall BZD deaths, street BZD deaths increased by over 1200% between 2015 (*n* = 26) and 2020 (*n* = 326). There were increasing proportions of street BZD deaths in each geographical region but was more marked in Northern Ireland. The proportion of individual BZDs seized at music festivals and submitted to WEDINOS largely reflected that of individual BZDs detected in deaths.

**Discussion and Conclusions:**

While deaths following street BZD use are increasing, most BZDs detected in deaths were prescribable BZDs that were often illicitly sourced. The types of BZD detected in post‐mortem samples, festival seizures and WEDINOS submissions has evolved over time to reflect changes in BZD prevalence on the illicit drug market.

**Key Points**

- The number of novel ‘street’ benzodiazepines (BZD) available is increasing.
- In Northern Ireland, street BZDs are a major driver of the drug‐related death trend.
- This trend is less apparent in England and Wales where non‐prescribed diazepam remains the biggest problem.
- Maintenance prescribing of BZDs may mitigate the risks of illicit BZDs, be that use of street BZDs or non‐prescribed diazepam, but needs careful consideration to reduce risk of diversion.

## 1 INTRODUCTION

‘Street’ benzodiazepines (BZD) are those not available on prescription via the National Health Service (NHS) and are classified more broadly as Novel Psychoactive Substances (NPS), a group of drugs that are created in a way to circumvent international control and scheduling as ‘legal highs’ [1]. Some street BZDs are licensed in other countries for clinical use (e.g., etizolam [2]), while others are research chemicals with no clinical use in humans (e.g., bromazolam [3]). These street BZDs consist of a closely related group of chemicals sharing BZD structure and pharmacology—allosteric potentiation of GABA at the GABAA receptor [4]. Similar to licensed BZDs available on prescription via the NHS, the effects of street BZDs are sedative and anxiolytic at low doses [5]. However, many street BZDs have not undergone the rigorous pharmacological testing required of licensed medicines, and this incomplete understanding of their pharmacology and toxicology can pose health risks to people who use them [3]. Indeed, from what we do understand many of these street BZDs possess higher potency at the GABA_A_ receptor and a shorter duration of action than diazepam [6, 7, 8, 9, 10, 11, 12], which can lead to repeated administration, reinforcing use behaviours and inducing dependence.

Drug‐related deaths (DRD) in England and Wales have risen year on year over the past decade [13]. In Scotland, this is paralleled by an eight‐fold increase in the involvement of street BZDs in DRDs [14]. Clinicians in Scotland have suggested that this rise in street BZD use may have occurred in response to the practice change of de‐prescribing BZDs, leading to a gap in the BZD market [14]. In England, BZD prescriptions have remained relatively stable in primary care [15]. However, in patients receiving opioid agonist treatment (i.e., methadone or buprenorphine for opioid dependence), the proportion receiving a concurrent BZD prescription has reduced from 42% in 1999 to 28% in 2014 [16], raising the possibility that a similar demand for illicit BZDs may arise in the English opioid agonist treatment population.

The rate of deaths where BZDs (both prescribable and ‘street’) have been detected in DRDs in England, Wales and Northern Ireland has doubled in the past 10 years [13] While various factors are at play, including that as overall DRDs in England and Wales have increased the number involving BZDs will have concordantly risen, it is possible that street BZD use is contributing to an increasing proportion of overall BZD deaths, as is the case in Scotland [14].

We therefore undertook an investigation into deaths where BZDs were detected at post‐mortem in England, Wales and Northern Ireland using data from the National Programme on Substance Use Mortality (NPSUM) to understand the relative contributions of prescribable and street BZDs to overall BZD deaths. We also sought to correlate trends in deaths where BZDs were detected with those in circulation on the illicit drug market by examining seizures of BZDs at music festivals and postal submissions to the drug testing service Welsh Emerging Drugs and Identification of Novel Substances (WEDINOS) (www.wedinos.org) in order to make inferences about toxicity.

## 2 METHODS

### 2.1 National Programme on Substance Use Mortality

#### 2.1.1 Dataset

Data were collated from case reports submitted to the NPSUM (formerly the National Programme on Substance Abuse Deaths [NPSAD]), which has received regular voluntary reports regarding drug‐related deaths from coroners since 1997. Cases included in this study comprise reports received from 78 of the 93 coronial jurisdictions (83.9%) in England, Wales and Northern Ireland. While the level of narrative detail provided in these reports varies between submitting coronial jurisdictions, all variables included in this analysis comprised complete data for all cases. A death is deemed drug‐related by coroners where drugs were considered contributory to the death occurring. Cases include deaths from prescription medications, recreational drugs, novel psychoactive substances, and intravenous drug use. Coroners investigate deaths resulting from a range of causes deemed to be unnatural; this includes violent and sudden deaths, unexplained deaths, deaths that occur before a patient comes out of anaesthetic, and deaths caused by industrial disease or poisoning. Toxicology tests are requested dependent upon individual case circumstances and at the discretion of the coroner and/or consulting pathologist.

The King's College London Biomedical and Health Sciences, Dentistry, Medicine and Natural and Mathematical Sciences Research Ethics Sub‐Committee re‐confirmed in August 2023 that NPSUM does not require research ethics committee review as all subjects are deceased.

#### 2.1.2 Case identification

Cases were identified as those reported to NPSUM by 1 November 2022 with post‐mortem detections of BZDs. All cases contained toxicology evidence confirming the presence of BZD parent molecules and/or metabolites in decedents' post‐mortem tissue(s). BZDs were classified as ‘prescribable’ or ‘street’ dependent upon their availability on prescription via the NHS according to the British National Formulary and NICE guidelines [17, 18] (Table 1). Where there were detections of prescribable BZDs that are active metabolites of other BZDs (e.g., temazepam and oxazepam as metabolites of diazepam) only the parent drug detection was included in the analysis. Toxicological evidence was used as the criteria to define qualifying cases rather than cause(s) of death as it is not uncommon for ambiguous drug‐related causes to be cited (e.g., multi‐drug toxicity, polydrug abuse), or environmental factors that caused death as a result of drug use (e.g., fall from a height) to be listed.

**TABLE 1:** Year of first detection.

Year	Prescribable BZD	Street BZD
1999–2010	Chlordiazepoxide	
	Clobazam	
	Clonazepam	
	Diazepam	Alprazolam*~^
	Lorazepam	Bromazepam*^
	Lormetazepam	Flunitrazepam*^
	Midazolam	Flurazepam*
	Nitrazepam	Triazolam*
	Oxazepam	
	Temazepam	
2011		Phenazepam*~^
2012		‐
2013		Etizolam*~^
		Pyrazolam*^
2014		Flubromazepam*^
2015		Diclazepam*^
		Flubromazolam*~^
2016		‐
2017		Clonazolam^ Nitrazolam^
2018		Ketazolam~
2019		Delorazepam*
		Flualprazolam*~^
2020		Adinazolam^ Cinolazepam*
		Flubroalprazolam*
		Meclonazepam*^
2021		Bromazolam*~
		Deschloroetizolam*^
		Estazolam*

*Note*: The following notations are used to demonstrate where street benzodiazepines (BZD) were detected in post‐mortem (*), music festival (~) and/or Welsh Emerging Drugs and Identification of Novel Substances (WEDINOS) (^) samples.

#### 2.1.3 Case analysis

IBM® SPSS software (Version 27) was used for case extraction, analysis and statistical tests.

Chi‐square tests were used to determine if associations between two categorical variables were significantly different (e.g., proportion of deaths where detected street vs. prescribable BZDs were implicated in causing death; proportion of street vs. prescribable BZDs were known drug users) and an independent two‐sample Student's *t*‐test was performed to compare the mean ages of those who died following street BZD versus prescribable BZD use. The significance threshold for all tests was set at *p* < 0.05.

The average time between death and coronial inquest conclusion for a drug‐related death is 7–10 months but can be longer. Further deaths occurring in 2021 are therefore anticipated to be reported to the NPSUM after the date of the data cut made for this analysis (1 November 2022). Based on jurisdiction reporting trends the total number of cases expected to be received by the NPSUM has been projected. While using date of death requires such a projection to be made (as opposed to date of reporting/registration), doing so enables comparison to Scottish data which is rapidly processed and reported.

Categorisation of BZDs detected at post‐mortem as having been prescribed, illicitly obtained or unknown in source were delineated based upon information provided in the NPSUM case reports derived from GP and hospital prescribing records.

Deprivation deciles (decile 1—most deprived; decile 10—least deprived) were determined by postcode matching the usual address of decedents with the English, Welsh and Northern Irish Indices of Deprivation calculators.

### 2.2 TICTAC/music festival tablet and capsule collection and analysis

#### 2.2.1 Sample collection

BZD tablets and capsules were collected from 22 music festivals which took place between 2017 and 2021 in England and Wales (note: no tablets were collected in 2020 as all music festivals were cancelled due to the COVID‐19 pandemic with fewer tablets collected in 2021 due to fewer festivals being held for the same reason; the names and locations of the festivals have not been disclosed in line with the confidentiality agreements signed allowing collation and testing of seized materials). The majority of the tablets were seized at entry gate searches, with a smaller number from voluntary surrender to amnesty bins.

#### 2.2.2 Gas‐chromatography mass‐spectrometry

Methanol (LC–MS grade) and t‐butyl‐methyl‐ether were purchased from Rathburn Chemicals (Walkerburn, Scotland). Tripelennamine and quinoline were used as internal standards to verify the retention times of the compounds under analysis and sensitivity of the instrument (Sigma‐Aldrich, Dorset, UK). Samples were qualitatively analysed using an Agilent 7890A GC with 5975C VL MSD (Agilent Technologies, Santa Clara, CA, USA), equipped with a split‐splitless injector and an HP5‐MS column (30 m × 0.25 mm, 0.25 μm film thickness). Agilent Mass Hunter software (version B07.06, Agilent Technologies) was used for data analysis.

#### 2.2.3 Samples preparation and method

For qualitative analysis, 1 mL of methanol was dispensed into a 1.5 mL polypropylene tube (Appleton Woods, Birmingham, UK) containing approximately 20 mg crushed tablet. Subsequently, the samples were vortexed for 30 min, then centrifuged for 1 min at 8000 rpm (6030 g). An extract volume of 100 μL was diluted in 1 mL of a t‐butyl‐methyl‐ether solution containing 100 μg/mL quinoline and tripelennamine. An aliquot of 1 μL was injected into the gas‐chromatography mass‐spectrometry using a 5:1 split ratio. The column was held at 80°C for 4 min and then ramped up at 40°C/min to 290°C for 10 min to a total run time of 19.25 min. For samples that produced no conclusive results, the column was held at 100°C for 4 min and then ramped up at 40°C/min to 310°C for 30 min to a total run time of 39.25 min. The MSD scanned a mass range of m/z 40 to 400 for each sample and all the GC and MS spectra obtained were compared against spectral libraries from Cayman, NIST and SWGDRUG alongside reference standard spectra from TICTAC.

### 2.3 WEDINOS data

The WEDINOS (www.wedinos.org) initiative enables people to anonymously submit drug samples for testing via post. People using the service are asked to provide the first half of the postcode from where they are submitting, the type of drug(s) they intended to purchase (if known), and to describe the effects of the drug if taken. The type of BZD detected in samples submitted to WEDINOS between 2017 and 2021 from England, Wales and Northern Ireland were recorded from their publicly available online webpages.

## 3 RESULTS

### 3.1 Post‐mortem BZD detections

**FIGURE 1:**
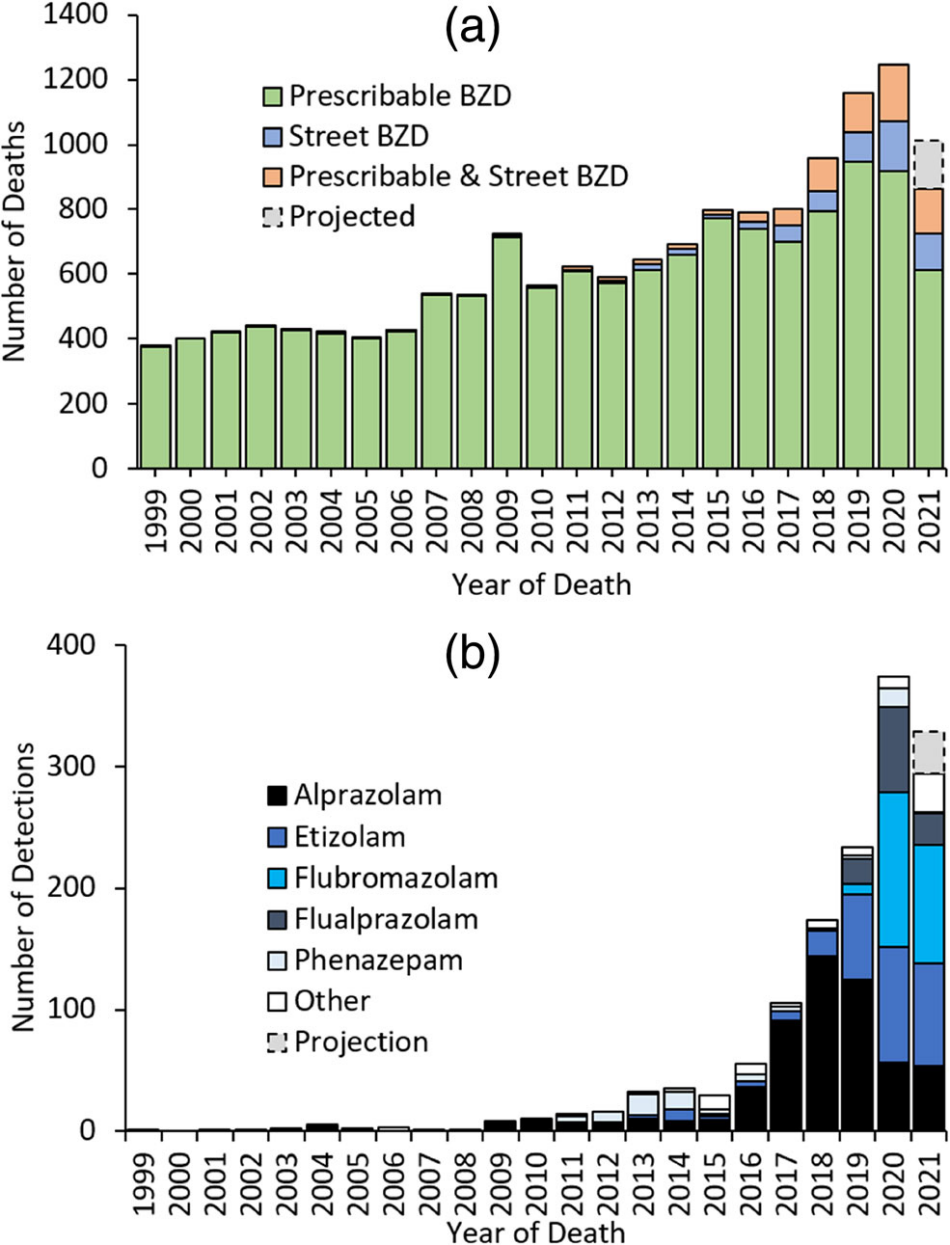
(a) Number of deaths reported to the National Programme on Substance Use Mortality where benzodiazepines (BZD) were detected at post‐mortem. As the average period of time between death and conclusion of coronial inquests is 7–10 months, further deaths from 2021 are anticipated to be received and have been projected based upon previous coronial jurisdiction reporting trends. (b) Number of detections of street BZDs in deaths reported to the National Programme on Substance Use Mortality. As the average period of time between death and conclusion of coronial inquests is 7–10 months, further deaths from 2021 are anticipated to be received and have been projected based upon previous coronial jurisdiction reporting trends. Other BZDs detected in 2021: bromazolam *n* = 16; flubromazepam *n* = 6; meclonazepam *n* = 3; bromazepam *n* = 2; flurazepam n = 1; triazolam n = 1; deschloroeitzolam n = 1; estazolam *n* = 1.

A total of 14,837 deaths with 16,208 detections of BZDs between 1999 and 2021 were extracted for analysis. Annual deaths following BZD use have more than doubled over the past decade (2010: 556 deaths; 2020: 1245 deaths; Figure 1). The overwhelming majority of BZDs were those available on prescription via the NHS, featuring in 96.2% of total BZD deaths (*n* = 14,267/14,837) and comprising 91.2% of total BZD detections (*n* = 14,776/16,208). However, when considering the prescribing status of decedents at the time of their death (known in 76.1% of deaths with a prescribable BZD detected, *n* = 10,857/14,267, from which there were 11,496 detections of prescribable BZDs), a larger proportion of these prescribable BZDs had been illicitly sourced (61.9% of detections, *n* = 7117/11,496) rather than legitimately prescribed to the deceased (38.1% of detections, *n* = 4379/11,496). Diazepam was by far the most commonly detected prescribable BZD that had been obtained without an active prescription, accounting for 77.3% of these detections (*n* = 5504/7117). By comparison, the contribution of street BZDs to overall BZD deaths and detections is relatively small (8.5% of total BZD deaths, *n* = 1264/14,837; 8.8% of total BZD detections, *n* = 1432/16,208; Figure 1).

**FIGURE 2:**
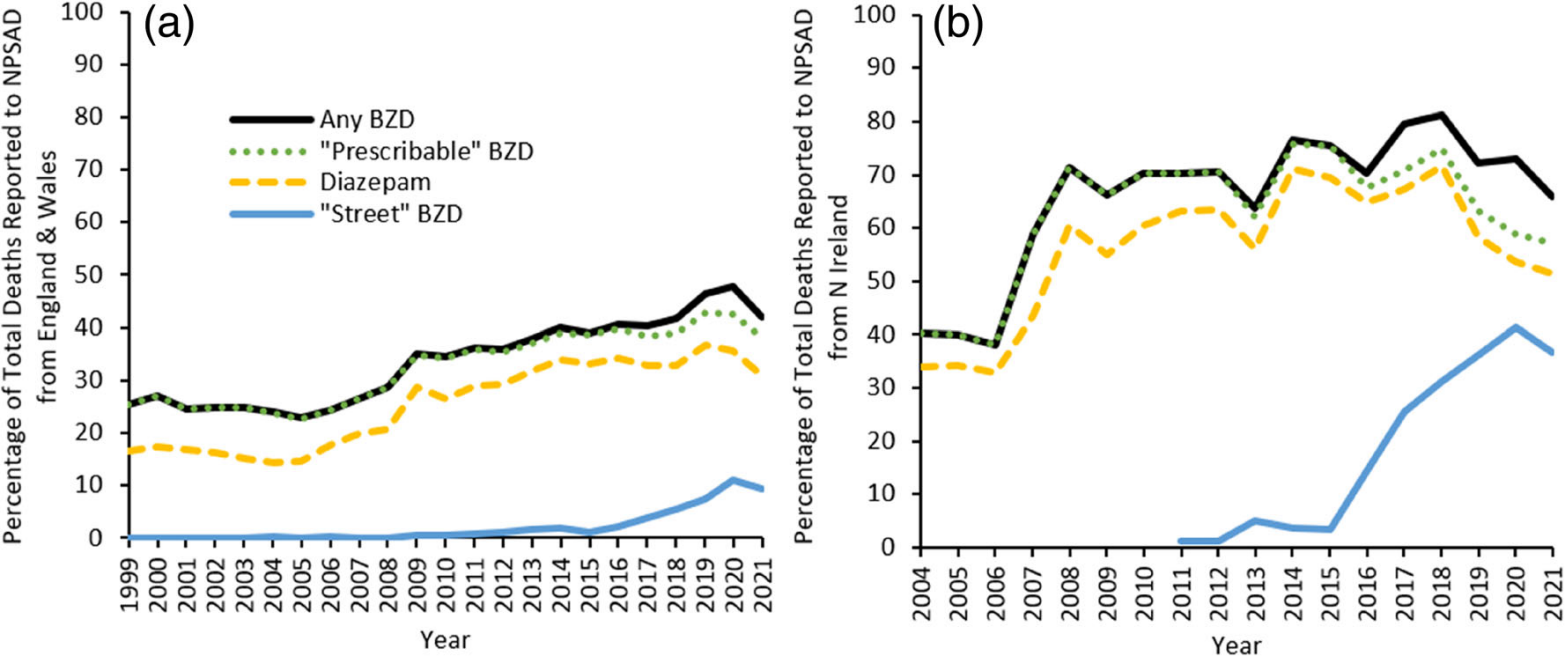
Proportion of deaths reported to the National Programme on Substance Use Mortality delineated by type of benzodiazepine (BZD) detected in (a) England and Wales and (b) Northern Ireland. Projected number of reports still to be received in 2021 have not been included.

Nevertheless, deaths following street BZD use increased by over 1200% between 2015 (*n* = 26 deaths, *n* = 29 detections) and 2020 (*n* = 326 deaths, *n* = 374 detections; Figure 1b). Indeed, when delineating by region, it is evident that there is an increasing proportion of deaths following street BZD use in England and Wales (Figure 2a), but this is even more marked in Northern Ireland (Figure 2b).

When detected at post‐mortem, prescribable BZDs were implicated in causing death in a significantly smaller proportion of cases than street BZDs (54.5% [*n* = 7781/14,267] and 70.5% [*n* = 891/1264] of deaths, respectively; *X* ^*2*^ *p* < 0.001). Rates of polydrug use were similarly high (99.2% [*n* = 14,152/14,257] and 99.4% [*n* = 1257/1264] of deaths, respectively; *X* ^*2*^ *p* > 0.05), as was co‐detection of opioids (85.6% [*n* = 12,068/14,267] and 84.9% [*n* = 1073/1264] of deaths, respectively; *X* ^*2*^ *p* > 0.05). Co‐detection of alcohol was comparatively less common, although a significantly higher proportion of deaths with prescribable BZDs had alcohol co‐detected than cases with street BZDs (38.1% [*n* = 5434/14,267] and 25.2% [*n* = 318/1264] of deaths, respectively; *X* ^*2*^ *p* < 0.001; cases where alcohol was attributed to likely post‐mortem production by the pathologist [≤10 mg/dL] [19] were excluded).

All prescribable BZDs were first detected between 1999 and 2010 (Table 1). In contrast only 5 of the 18 street BZDs were detected in this period, with 8 detected for the first time between 2019 and 2021 (Table 1): a shifting pattern is evident, with alprazolam and phenazepam the most commonly detected street BZDs until 2018, after which etizolam, flubromazolam and flualprazolam detections prevail (Figure 1b).

### 3.2 Decedent demographics

**FIGURE 3:**
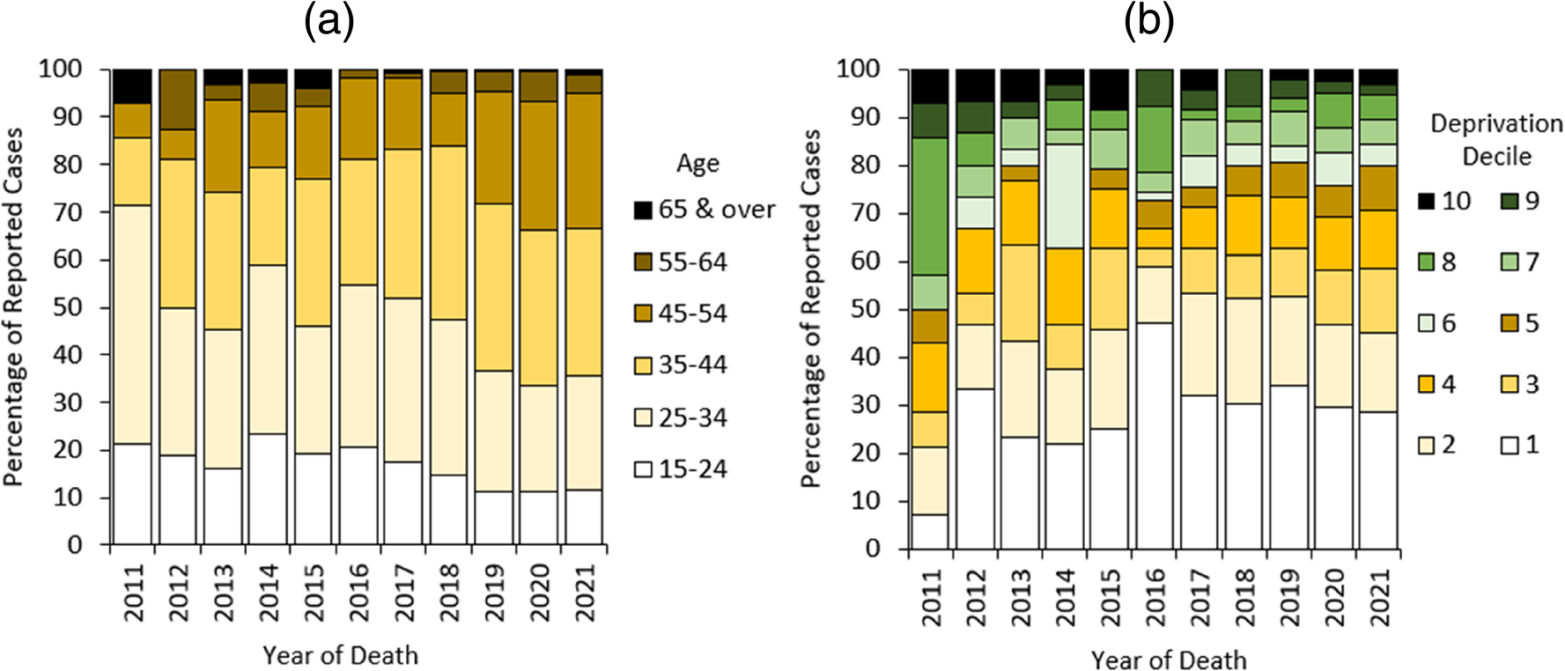
(a) Age group over time of people who died following street benzodiazepine (BZD) use. (b) Decile of deprivation over time of usual address of people who died following street BZD use. These indices are calculated considering the following variables: income, employment, education, skills and training, health and disability, crime, barriers to housing and services and living environment. Areas in Decile 1 are considered the most deprived in the country, and those in Decile 10 the least deprived.

People who died following street BZD use were more likely than those who had used prescribable BZDs to have been male (82.8% of street BZD cases, *n* = 1047/1264; 72.9% of prescribable BZD cases, *n* = 10,402/14,267; *X* ^*2*^ *p* < 0.001), younger at the time of their death (mean age street BZD cases 37.4 [±11.0]; prescribable BZD cases 40.5 [±12.3]; Student's *t* test *p* < 0.05) and have had a known history of substance use (where this information was provided: street BZD cases 90.9%, *n* = 888/977; prescribable BZD cases 81.1%, *n* = 9222/11,374; *X* ^*2*^ *p* < 0.001). Furthermore, the average age of people who died following street BZD use increased over time (mean age 2011: 34.4 ± 16.9; 2021 38.8 ± 10.6), and they were increasingly living in the more deprived areas of the country (mean percent of decedents living in the most deprived decile 2011–2013: 21.3%; 2019–2021 30.8%; Figure 3).

### 3.3 BZDs seized from music festivals and submitted to WEDINOS

There were 461 seizures of BZD tablets and capsules (total 4484.5 tablets and capsules) made at the 22 music festivals held in England and Wales included in this study (2017: 173 seizures, 1398 tablets and capsules; 2018: 116 seizures, 1487 tablets and capsules; 2019: 147 seizures, 1393 tablets and capsules; 2021: 25 seizures, 206.5 tablets and capsules; note: no seizures were made in 2020 as music festivals were not held due to the COVID‐19 pandemic, with fewer festivals hosted in 2021 for the same reason). Analysis detected 14 different BZDs. Diazepam was the most commonly seized BZD (41.9% of seizures, *n* = 193/461), followed by alprazolam (30.4%, *n* = 140/461) and etizolam (20.0%, *n* = 92/461). The other 11 BZDs were detected in much smaller numbers and together comprised the remaining 7.8% (*n* = 36/461).

There were 3554 submissions to WEDINOS between 2017 and 2021 where a BZD was detected upon analysis (submissions in 2017: 146; 2018: 306; 2019: 716; 2020: 948; 2021: 1438). Analysis detected 23 different BZDs. Diazepam was the most commonly detected BZD (31.4% of submissions, *n* = 1116/3554) followed by etizolam (20.2%, *n* = 719/3554), alprazolam (13.8%, *n* = 492/3554), flubromazolam (11.4%, *n* = 405/3554), and flualprazolam (10.9%, *n* = 386/3554). The other 18 BZDs were detected in much smaller numbers and together comprised the remaining 12.3% (*n* = 436/3554).

Ketazolam, a street BZD detected in one seizure (10 tablets) in 2018, had not been detected in any deaths reported to the NPSUM nor in any submission to WEDINOS. Comparatively there were post‐mortem detections and/or WEDINOS submissions of 16 street BZDs which were not identified in any of the seizures (Table 1).

**FIGURE 4:**
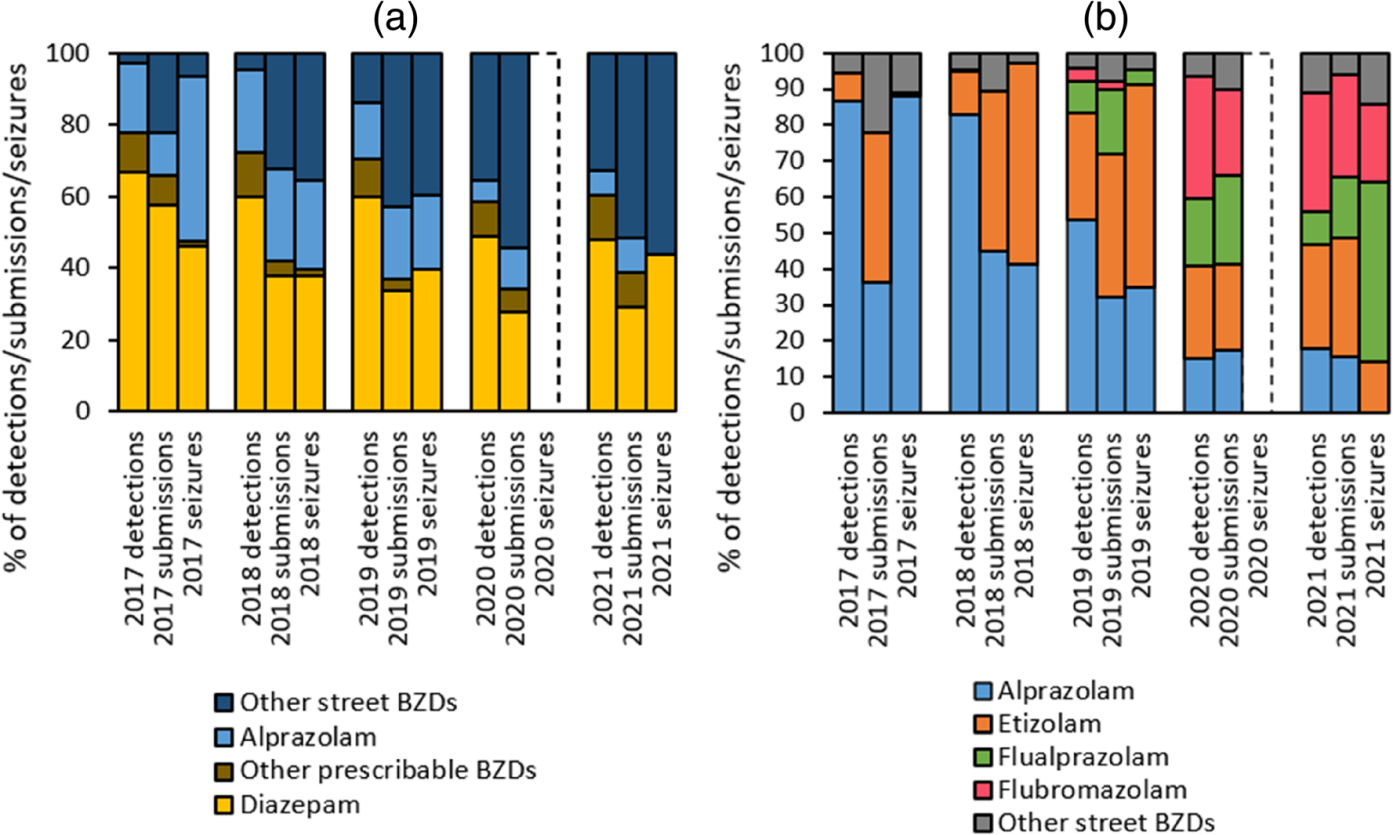
(a) Comparative proportions of overall benzodiazepine (BZD) post‐mortem detections, WEDINOS submissions, and seizures at music festivals delineated by year. Other prescribable BZDs: chlordiazepoxide, clobazam, clonazepam, lorazepam, lormetazepam, midazolam, nitrazepam, oxazepam, temazepam. Other street BZDs: adinazolam, bromazepam, bromazolam, cinolazepam, clonazolam, delorazepam, deschloroetizolam, diclazepam, estazolam, etizolam, flualprazolam, flubroalprazolam, flubromazepam, flubromazolam, flunitrazepam, flurazepam, ketazolam, meclonazepam, nitrazolam, phenazepam, pyrazolam, triazolam. (b) Comparative proportion of street BZD post‐mortem detections, WEDINOS submissions, and seizures at music festivals delineated by year. Other street BZDs: adinazolam, bromazepam, bromazolam, cinolazepam, clonazolam, delorazepam, deschloroetizolam, diclazepam, estazolam, flubroalprazolam, flubromazepam, flunitrazepam, flurazepam, ketazolam, meclonazepam, nitrazolam, phenazepam, pyrazolam, triazolam.

Over time, the proportion of both prescribable and street BZDs seized from music festivals and submitted to WEDINOS largely mirrors that of BZDs detected in deaths with those of diazepam and alprazolam decreasing and other street BZDs increasing (Figure 4a). When examining the street BZDs alone, alprazolam and etizolam were dominant in circulation pre‐2020, with a greater variety of newer street BZDs both seized, detected at post‐mortem, and submitted to WEDINOS in 2021 (Figure 4b).

## 4 DISCUSSION

To our knowledge, this is the largest report on deaths following street BZD use to date. Following the first reported fatality which occurred in 1999, the number of deaths with street BZD detections gradually increased from a steady baseline from 2007 to 2017, and more markedly thereafter.

However, despite the intended focus of this analysis on street BZDs, it is immediately apparent that diazepam is the single most prevalent BZD detected in DRDs, music festival seizures, and WEDINOS submissions from England, Wales and Northern Ireland. Deaths following diazepam use are attributable to both prescribed diazepam and that which was illicitly procured, the latter of which is likely a combination of diazepam diverted from prescriptions [20] and counterfeit supplies [21, 22].

### 4.1 There are regional disparities in street BZD deaths

In England and Wales, diazepam continues to be the most commonly detected BZD, and most closely parallels the increase in overall DRDs. Contrastingly, the trend in Northern Ireland more closely resembles that observed in Scotland where street BZDs have accounted for a greater proportion of total BZD post‐mortem detections since 2016 [14]. This may relate to the proximity of Northern Ireland to illegal drug laboratories operating out of Scotland [23] but there are further reasons why the situation in Northern Ireland is different. Northern Ireland has historically had higher BZD prescribing rates than the rest of the United Kingdom [24], declining in the 1980s but increasing once more to reach a higher plateau in the 1990s [25] and persist into the 2000s [26]. This disparity has been attributed to trauma related to the Troubles, decades long sectarian violence which occurred up to the Good Friday Agreement in 1994 [26, 27]. Thirty years on, people in Northern Ireland continue to suffer from higher rates of mental ill health than the rest of the United Kingdom, with the prevalence of post‐traumatic stress disorder the highest in all countries which have produced such estimates, including the United States and Western Europe [27, 28, 29].

McAuley et al. have hypothesised that a potential driver for the increase in DRDs in Scotland is the widespread de‐prescribing of BZDs, with the resultant void being filled by street BZDs that may be more toxic than diazepam [14]. In England, BZD prescribing has remained stable in primary care (but decreased in patients receiving opioid agonist treatment—see BZD deaths relate to polysubstance use section) [15, 16]. As the trajectory of deaths following street BZD use in England and Wales does not closely track the trajectory of overall DRDs as it does in Scotland, the argument made for maintenance prescribing of BZDs as a harm reduction measure against street BZD use in Scotland [14] does not appear to hold in England and Wales, but may do for Northern Ireland. Further, given the high prevalence of diazepam in DRD in England and Wales, maintenance prescribing of BZDs to curb illicit BZD use would need to consider the potential for diversion and whether initiating patients on a daily supervised consumption regime of such prescriptions is warranted to mitigate the risk of DRDs, as is done with the opioid agonist therapy methadone [30, 31].

### 4.2 Post‐mortem toxicology data reflects the drugs market in street BZDs

The wide variety of street BZDs detected in 2021 highlights the rapid pace at which these drugs are substituted on the illicit market. The European Union Early Warning System reported 1012 seizures of street BZDs in 2020, amounting to more than 65,000 tablets and capsules; however, there has been an overall decline in seizures following a peak in 2015 [32]. Despite this decline, deaths following street BZD use continued to rise each year to 2019. The street BZDs detected in the greatest number of deaths—alprazolam, etizolam, flubromazolam and flualprazolam—largely mirrors that of street BZDs seized at music festivals and submitted to WEDINOS, suggesting that the street BZDs in widest circulation are the ones that have the greatest contribution to death, rather than representing disproportionate toxicity. These street BZDs are, however, all more potent than diazepam [10, 11, 12], which may account for their increased rate of implication in causing death. In addition, these data suggest that while different user demographics will be represented by those dying following drug use, those who attend music festivals, and the wide variety of people who submit samples to WEDINOS (both users of drugs, dealers of drugs, and those who have found drugs [e.g., nightclub staff, hostel workers]), the types of BZDs sold are not tailored to the user group but rather dictated by those which are available on the illicit drug market.

Of the street BZDs seized at music festivals, ketazolam was the only one not detected in any post‐mortem samples or WEDINOS submissions. Although not licensed in the United Kingdom, ketazolam is regarded as a safe medication [33], meaning one of four things: either it is safer to consume than other street BZDs, the seizure in 2018 confiscated all ketazolam and none is left in circulation, there is little ketazolam available on the illicit market, or it is not routinely screened for in post‐mortem toxicology testing.

### 4.3 BZD deaths relate to polysubstance use

Previous mortality data for street BZDs show that death is unlikely to occur as the result of the single drug taken, but rather as a result of mixed drug toxicity [34, 35, 36, 37, 38]. Indeed, polysubstance use heightens the risk of overdose through increased respiratory depression and sedation [39]. Although alcohol and BZD co‐use act synergistically as central nervous system depressants (CNS) [40], there was a relatively low proportion of decedents with alcohol co‐detected at post‐mortem in this study. Rather, decedents used other CNS depressants—predominantly opioids—more frequently.

Our finding that 70% of decedents have a known history of illicit substance use reflects known patterns of substance use in addictions treatment populations. BZD use among people undergoing opioid agonist treatment is common, with many patients either initiating BZD use after entering opioid agonist treatment or increasing their use during treatment [41]. This is concerning because people on opioid agonist treatment concurrently taking BZDs are more likely to have poorer clinical outcomes, such as increased opioid toxicity and mortality [16, 42]. The use of more potent street BZDs with other CNS depressants is of additional concern, although it is possible that due to the high adulteration rates of street BZDs in tablets sold as prescribable BZDs [43, 44, 45], a number of decedents were likely unknowingly taking a drug more harmful than expected.

### 4.4 Deaths following street BZD use are occurring in a majority male, increasingly aged and deprived population

It is well established that drug use and DRDs are more predominant in men [13]. There are likely several different reasons for this, including that men are more likely to engage in risky behaviour [46], drug use in women is more stigmatised [47], and drugs (and alcohol) are more readily available to men [47]. Decedents with street BZD detections were increasingly aged and living in more deprived areas over time. This may relate to the putative consequence of the *Psychoactive Substances Act* (2016) whereby a shift in the sale of NPS from ‘head shops’ into more underground markets led to subsequent decreases in deaths in younger affluent recreational drug users and increases in deaths occurring in people with established dependent drug use living in the most deprived deciles [8, 48]. As the illicit BZD market operates on the streets as well as via the internet, and street dealers operate in the more deprived areas, these drugs are more accessible to vulnerable populations [49]. Individuals living in the more deprived areas of England are at a significantly greater risk of avoidable mortality from alcohol and drug‐related disorders [13]. This has similarly been described in drug‐related deaths in Scotland [50].

### 4.5 Limitations

NPSUM is reported to voluntarily by coroners. Therefore, while the data in this study are likely indicative of the overall trends in deaths following street BZD use in England, Wales and Northern Ireland, they are likely an under‐representation of the true number of deaths which have occurred. However, as not all deaths are reported to a coroner for investigation, and those which are do not always undergo rigorous toxicology testing which includes drug screening panels for NPS such as street BZDs, even with a 100% coronial reporting rate such a sample would still be only a representative proportion of the actual trend.

The population of people who suffer a DRD and those who attend music festivals are likely two distinct populations of largely dependent and recreational drug users, respectively. While the data presented here from these populations are largely analogous, this may account for some of the differences seen (e.g., lack of ketazolam detections in post‐mortem samples). However, users from these populations and others (e.g., dealers of drugs, people who work in environments where drugs are found [nightclubs, hostels]) are likely represented by those who have submitted to WEDINOS, with WEDINOS data further corroborating similarity in the illicit drug supply (and a lack of ketazolam submissions).

## 5 CONCLUSIONS

These data demonstrate that while street BZDs account for an increasing proportion of overall deaths following BZD use in Northern Ireland, this is not the case for England and Wales where deaths following BZD use remain predominantly due to use of non‐prescribed diazepam. Maintenance prescribing of BZDs may therefore be an appropriate harm reduction measure to safeguard against the risks posed by illicit BZDs, be this due to street BZD or counterfeit licensed BZD use, but needs careful consideration to reduce risk of diversion. The types of BZD detected in post‐mortem samples, festival seizures and WEDINOS submissions has evolved over time to reflect changes in BZD prevalence on the illicit drug market.

## AUTHOR CONTRIBUTIONS

Kirsten L Rock analysed the data, wrote the manuscript, and reviewed the final version of the manuscript. Anca Frinculescu analysed the data and reviewed the final version of the manuscript. Trevor Shine supported the data collection, analysed the data, and reviewed the final version of the manuscript. Nicola J Kalk conceived the research, wrote the manuscript, and reviewed the final version of the manuscript. Caroline S Copeland supported the data collection, conceived the research, and reviewed the final version of the manuscript.

## CONFLICT OF INTEREST STATEMENT

Kirsten L Rock is supported by a PhD studentship from the Society for the Study of Addiction. Nicola J Kalk is supported by the NIHR Biomedical Research Centre for Mental Health at South London and Maudsley NHS Foundation Trust and King's College London. Caroline S Copeland is a co‐opted member of the Advisory Council for the Misuse of Drugs (ACMD) Novel Psychoactive Substances (NPS) Sub‐Committee. Anca Frinculescu and Trevor Shine have nothing to declare.

## Data Availability

The data that support the findings of this study are available on request from the corresponding author. The data are not publicly available due to privacy or ethical restrictions.
